# (*E*)-3-Ferrocenyl-1-(2-hy­droxy­phen­yl)-prop-2-en-1-one

**DOI:** 10.1107/S1600536810023378

**Published:** 2010-06-23

**Authors:** C. Valdebenito, M. T. Garland, M. Fuentealba, C. A. Escobar

**Affiliations:** a Universidad Andres Bello, Departamento de Ciencias Químicas, Santiago, Chile; b Laboratorio de Cristalografía, Departamento de Física, Facultad de Ciencias Físicas y Matemáticas, Universidad de Chile, Santiago, Chile

## Abstract

The mol­ecular structure of the title compound, [Fe(C_5_H_5_)(C_14_H_11_O_2_)] consists of a ferrocenyl and 2-hy­droxy­phenyl group linked through the prop-2-en-1-one spacer and is stabilized by an intra­molecular O—H⋯O hydrogen bond between the hydroxyl and the carbonyl groups.

## Related literature

For biological activity of chalcones, see: Liu *et al.* (2001 ▶); Rao *et al.* (2004 ▶); Wu *et al.* (2002 ▶, 2006 ▶); Xiang *et al.* (2006 ▶); Zsoldos-Mady *et al.* (2006 ▶). For their non-linear optical properties, see: Shettigar *et al.* (2006 ▶). For electro-active fluorescent materials, see: Belavaux-Nicot *et al.* (2005 ▶). For related structures, see: Escobar *et al.* (2008 ▶). For metallocene derivatives, see: Kudar *et al.* (2005 ▶).
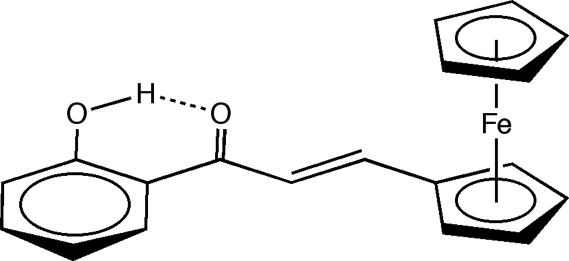


## Experimental

### 

#### Crystal data

[Fe(C_5_H_5_)(C_14_H_11_O_2_)]
*M* *_r_* = 332.17
Monoclinic, 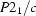

*a* = 10.8264 (12) Å
*b* = 12.0358 (13) Å
*c* = 11.8150 (13) Å
β = 103.839 (2)°
*V* = 1494.9 (3) Å^3^
*Z* = 4
Mo *K*α radiation
μ = 1.01 mm^−1^
*T* = 298 K
0.25 × 0.19 × 0.15 mm

#### Data collection

Bruker SMART CCD area-detector diffractometer
Absorption correction: multi-scan (*SABABS*; Bruker, 2000 ▶) *T*_min_ = 0.741, *T*_max_ = 0.859
11654 measured reflections
3319 independent reflections
2734 reflections with *I* > 2σ(*I*)
*R*_int_ = 0.016

#### Refinement

*R*[*F* ^2^ > 2σ(*F* ^2^)] = 0.043
*wR*(*F* ^2^) = 0.120
*S* = 1.02
3319 reflections
199 parameters
16 restraints
H-atom parameters constrained
Δρ_max_ = 0.69 e Å^−3^
Δρ_min_ = −0.21 e Å^−3^

### 

Data collection: *SMART* (Bruker, 2001 ▶); cell refinement: *SAINT* (Bruker, 2000 ▶); data reduction: *SAINT*; program(s) used to solve structure: *SHELXS97* (Sheldrick, 2008 ▶); program(s) used to refine structure: *SHELXL97* (Sheldrick, 2008 ▶); molecular graphics: *OLEX2* (Dolomanov *et al.*, 2009 ▶); software used to prepare material for publication: *OLEX2*.

## Supplementary Material

Crystal structure: contains datablocks I. DOI: 10.1107/S1600536810023378/rk2207sup1.cif

Structure factors: contains datablocks I. DOI: 10.1107/S1600536810023378/rk2207Isup2.hkl

Additional supplementary materials:  crystallographic information; 3D view; checkCIF report

## Figures and Tables

**Table 1:** Hydrogen-bond geometry (Å, °)

*D*—H⋯*A*	*D*—H	H⋯*A*	*D*⋯*A*	*D*—H⋯*A*
O2—H2*A*⋯O1	0.82	1.79	2.523 (3)	148

**Fig. 1.**
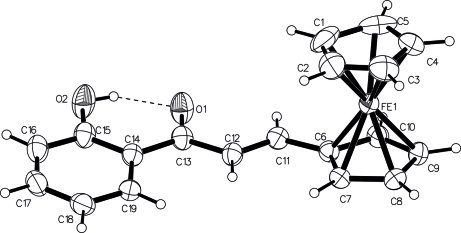
The molecular structure of title compound with the atom numbering scheme. Displacement ellipsoids are presented at 30% probability level. H atoms are shown as a small spheres of arbitrary radius.

## supplementary crystallographic information

### Comment

Chalcones are a family of aromatic ketones bearing two aromatic groups bridged together trough a central prop-2-en-1-one core. They have a wide range of applications covering from materials with various biological activities (Xiang *et al.*,  2006; Wu *et al.*,  2002, 2006; Liu *et al.*,  2001; Zsoldos-Mady *et al.*, 2006) to non-linear optics (Shettigar *et al.*,  2006), including also electroactive fluorescent materials (Belavaux-Nicot *et al.*, (2005). Ferrocene containing chalcones are also useful precursors for the synthesis of more complex heterocyclic metallocene derivatives (Kudar *et al.*, (2005). Typically this kind of precursors are prepared trough a *Claisen Shmidt* condensation, reacting a ketone or an aldehyde with the appropriated ferrocene substituted aldehyde or ketone depending on the position were the ferrocenyl substituent is required.

For the case of 1-(2'-hydroxyphenyl)-substituted prop-2-en-1-ones (as the title compound) the presence of an intense H-bonding has been previously described (Escobar *et al.*, 2008) this structural characteristic has been recognized to play a key role in its biological activity (Rao *et al.*, (2004).

The molecular structure of the title compound is composed by the ferrocenyl moiety and the 2'-hydroxyphenyl group joined by the organic prop-2-en-1-one spacer.

The dihedral angle between the two aromatic rings joined by the conjugated organic spacer is 12.01 (9)°. The distances between the C_5_H_5_- and C_5_H_4_-ring centroids to the iron atom are 1.653 (1)Å and 1.648 (1)Å, respectively. Both cyclopentadienyl rings of the ferrocenyl group are coplanar with a dihedral angle 1.6 (1)°. These metrical parameters are typical of the η^5^···Fe···η^5^ coordination of the ferrocenyl moiety.

The main feature of the structure is an intramolecular hydrogen bond O—H···O between the hydroxyl and the carbonyl group forming a six-membered ring helping the molecular stabilization, this characteristic has been previously observed in other 2'-hydroxy chalcones (Escobar *et al.*, 2008). This intramolecular bond leads the carbonyl group to display an *S*-*cis*-configuration in relation to the double bond. The double bond distance C═C is 1.325 (3)Å and exhibits a *cis*-conformation.

Finally, no intermolecular hydrogen bonds are observed in the crystalline packing of title compound.

### Experimental

The title compound was prepared as follows: a solution of potassium hydroxide (2 g in 30 ml methanol) was added to a mixture of ferrocenecarboxaldehyde (0.6 g 4.4 mmol) and 2-hydroxyacetophenone (0.94 g 4.4 mmol). The mixture was allowed to react for three days under continuous stirring. Then, methanol was evaporated in a rotatory evaporator and the crude reaction mixture was submitted to column chromatography (silica gel 60, Ethyl acetate: Hexane = 1: 20 *v*/*v*). The combined fractions containing the title compound were evaporated *in vacuo*, redissolved in methanol, and allowed to crystallize, to give violet crystals (65%), mp. 405.3–406.6 °C. IR (KBr) cm^-1^: 3456 (OH), 3105 (C—H), 3086 (C—H), 1630 (C═O). ^1^H NMR (CDCl_3_, 400 MHz): δ 4.22 (5*H*, s, C_5_H_5_), 4.56 (2*H*, s, –C_5_H_4_), 4.65 (2*H*, s, –C_5_H_4_), 6.95 (1*H*, t, J = 7.2 Hz, H_arom_), 7.26 (1*H*, d, J = 15 Hz, ═CH), 7.45 (1*H*, t, J = 7.2 Hz, H_arom_), 7.88 (1*H*, d, J = 9.1 Hz, H_arom_), 7.92 (1*H*, d, J = 15 Hz, ═CH), 13.08 (1*H*, s, OH). ^13^C NMR (CDCl_3_, 400 MHz): δ: 31.34, 69.51, 69.71, 70.36, 72.27, 117.15, 119.00, 119.10, 129.79, 136.30, 148.36, 164.03, 193.17.

### Refinement

The hydrogen atoms positions were calculated after each cycle of refinement with using a riding model with C—H distances in the range 0.93Å and 0.98Å. The *U*_iso_(H) values were set equal to 1.2*U*_eq_(C). The exception were the hydroxyl hydrogen atom which were located in the Fourier and then refined with the O—H distance constrained to be 0.82Å and the *U*_eq_ free to refine.

### Crystal data

**Table:**

[Fe(C_5_H_5_)(C_14_H_11_O_2_)]	*F*(000) = 688
*M**_r_* = 332.17	*D*_x_ = 1.476 Mg m^−^^3^
Monoclinic, *P*2_1_/*c*	Mo *K*α radiation, λ = 0.71073 Å
Hall symbol: -P 2ybc	Cell parameters from 4215 reflections
*a* = 10.8264 (12) Å	θ = 2.5–25.7°
*b* = 12.0358 (13) Å	µ = 1.01 mm^−^^1^
*c* = 11.8150 (13) Å	*T* = 298 K
β = 103.839 (2)°	Prism, violet
*V* = 1494.9 (3) Å^3^	0.25 × 0.19 × 0.15 mm
*Z* = 4	

### Data collection

**Table:**

Bruker SMART CCD area-detector diffractometer	3319 independent reflections
Radiation source: fine-focus sealed tube	2734 reflections with *I* > 2σ(*I*)
Parallel, graphite	*R*_int_ = 0.016
φ– and ω–scans	θ_max_ = 27.8°, θ_min_ = 1.9°
Absorption correction: multi-scan (*SABABS*; Bruker, 2000)	*h* = −14→13
*T*_min_ = 0.741, *T*_max_ = 0.859	*k* = −15→15
11654 measured reflections	*l* = −15→15

### Refinement

**Table:**

Refinement on *F*^2^	Primary atom site location: structure-invariant direct methods
Least-squares matrix: full	Secondary atom site location: difference Fourier map
*R*[*F*^2^ > 2σ(*F*^2^)] = 0.043	Hydrogen site location: inferred from neighbouring sites
*wR*(*F*^2^) = 0.120	H-atom parameters constrained
*S* = 1.02	*w* = 1/[σ^2^(*F*_o_^2^) + (0.0672*P*)^2^ + 0.5241*P*] where *P* = (*F*_o_^2^ + 2*F*_c_^2^)/3
3319 reflections	(Δ/σ)_max_ = 0.001
199 parameters	Δρ_max_ = 0.69 e Å^−^^3^
16 restraints	Δρ_min_ = −0.21 e Å^−^^3^

### Special details

**Table:**

Experimental. The data collection nominally covered a full sphere of reciprocal space by a combination of 5 sets of ω-scans each set at different φ- and/or 2θ-angles and each scan (10.00 s exposure) covering -0.300° in ω. The crystal to detector distance was 6.275 cm.
Geometry. All s.u.'s (except the s.u. in the dihedral angle between two l.s. planes) are estimated using the full covariance matrix. The cell s.u.'s are taken into account individually in the estimation of s.u.'s in distances, angles and torsion angles; correlations between s.u.'s in cell parameters are only used when they are defined by crystal symmetry. An approximate (isotropic) treatment of cell s.u.'s is used for estimating s.u.'s involving l.s. planes.
Refinement. Refinement of *F*^2^ against ALL reflections. The weighted *R*-factor *wR* and goodness of fit *S* are based on *F*^2^, conventional *R*-factors *R* are based on *F*, with *F* set to zero for negative *F*^2^. The threshold expression of *F*^2^ > σ(*F*^2^) is used only for calculating *R*-factors(gt) *etc*. and is not relevant to the choice of reflections for refinement. *R*-factors based on *F*^2^ are statistically about twice as large as those based on *F*, and *R*-factors based on ALL data will be even larger.

### Fractional atomic coordinates and isotropic or equivalent isotropic displacement parameters (Å^2^)

**Table:**

	*x*	*y*	*z*	*U*_iso_*/*U*_eq_	
Fe1	0.61269 (3)	0.33755 (3)	0.17323 (3)	0.05064 (15)	
C1	0.5167 (5)	0.2765 (4)	0.2865 (3)	0.1144 (12)	
H1	0.5238	0.2999	0.3673	0.137*	
C2	0.5826 (4)	0.1914 (3)	0.2476 (4)	0.1009 (10)	
H2	0.6489	0.1457	0.2973	0.121*	
C3	0.5421 (3)	0.1817 (3)	0.1306 (3)	0.0842 (8)	
H3	0.5750	0.1285	0.0823	0.101*	
C4	0.4497 (3)	0.2579 (3)	0.0903 (3)	0.0835 (8)	
H4	0.4060	0.2682	0.0082	0.100*	
C5	0.4286 (4)	0.3200 (3)	0.1808 (5)	0.1027 (10)	
H5	0.3656	0.3793	0.1767	0.123*	
C6	0.7415 (2)	0.44963 (19)	0.2599 (2)	0.0506 (5)	
C7	0.8045 (2)	0.3637 (2)	0.2124 (2)	0.0532 (5)	
H7	0.8698	0.3134	0.2565	0.064*	
C8	0.7558 (2)	0.3642 (2)	0.0898 (2)	0.0584 (6)	
H8	0.7808	0.3132	0.0345	0.070*	
C9	0.6634 (2)	0.4489 (2)	0.0607 (2)	0.0596 (6)	
H9	0.6137	0.4670	−0.0179	0.072*	
C10	0.6540 (2)	0.5021 (2)	0.1649 (2)	0.0556 (6)	
H10	0.5972	0.5640	0.1711	0.067*	
C11	0.7555 (2)	0.4760 (2)	0.3823 (2)	0.0545 (6)	
H11	0.6998	0.5281	0.4005	0.065*	
C12	0.8411 (2)	0.4321 (2)	0.4701 (2)	0.0554 (6)	
H12	0.8994	0.3811	0.4543	0.066*	
C13	0.8463 (2)	0.4620 (2)	0.5922 (2)	0.0562 (6)	
C14	0.9390 (2)	0.40822 (19)	0.6881 (2)	0.0531 (5)	
C15	0.9355 (3)	0.4298 (2)	0.8046 (2)	0.0617 (6)	
C16	1.0187 (3)	0.3761 (3)	0.8961 (2)	0.0715 (8)	
H16	1.0132	0.3887	0.9724	0.086*	
C17	1.1078 (3)	0.3054 (3)	0.8743 (3)	0.0740 (8)	
H17	1.1643	0.2709	0.9361	0.089*	
C18	1.1157 (3)	0.2841 (3)	0.7616 (3)	0.0693 (7)	
H18	1.1776	0.2358	0.7479	0.083*	
C19	1.0325 (2)	0.3339 (2)	0.6694 (2)	0.0567 (6)	
H19	1.0380	0.3184	0.5937	0.068*	
O1	0.7687 (2)	0.52989 (18)	0.61393 (17)	0.0793 (6)	
O2	0.8498 (3)	0.5003 (2)	0.83039 (17)	0.0900 (7)	
H2A	0.8062	0.5269	0.7700	0.135*	

### Atomic displacement parameters (Å^2^)

**Table:**

	*U*^11^	*U*^22^	*U*^33^	*U*^12^	*U*^13^	*U*^23^
Fe1	0.0501 (2)	0.0550 (2)	0.0459 (2)	−0.00450 (14)	0.00977 (15)	0.00348 (13)
C1	0.141 (3)	0.145 (3)	0.0747 (14)	−0.082 (2)	0.0616 (16)	−0.0231 (17)
C2	0.099 (2)	0.099 (2)	0.0958 (17)	−0.0357 (15)	0.0057 (16)	0.0430 (17)
C3	0.081 (2)	0.0678 (17)	0.1030 (17)	−0.0195 (12)	0.0212 (16)	−0.0147 (15)
C4	0.0549 (15)	0.109 (2)	0.0805 (15)	−0.0214 (12)	0.0050 (12)	0.0021 (14)
C5	0.074 (2)	0.097 (2)	0.159 (3)	−0.0151 (14)	0.071 (2)	−0.0085 (19)
C6	0.0511 (12)	0.0509 (12)	0.0483 (12)	−0.0034 (10)	0.0088 (10)	0.0028 (10)
C7	0.0466 (12)	0.0587 (13)	0.0531 (13)	0.0017 (10)	0.0098 (10)	0.0019 (10)
C8	0.0563 (14)	0.0698 (15)	0.0527 (13)	0.0011 (12)	0.0202 (11)	−0.0004 (12)
C9	0.0608 (14)	0.0674 (16)	0.0486 (13)	−0.0015 (12)	0.0093 (11)	0.0130 (11)
C10	0.0566 (13)	0.0506 (13)	0.0579 (14)	0.0029 (11)	0.0102 (11)	0.0079 (10)
C11	0.0583 (14)	0.0496 (12)	0.0553 (13)	−0.0034 (11)	0.0131 (11)	−0.0032 (10)
C12	0.0608 (14)	0.0560 (13)	0.0484 (13)	−0.0028 (11)	0.0110 (11)	−0.0026 (10)
C13	0.0636 (15)	0.0491 (12)	0.0536 (13)	−0.0032 (11)	0.0094 (11)	−0.0047 (10)
C14	0.0602 (14)	0.0474 (12)	0.0492 (12)	−0.0122 (10)	0.0082 (10)	−0.0038 (10)
C15	0.0763 (17)	0.0538 (14)	0.0520 (13)	−0.0079 (13)	0.0096 (12)	−0.0074 (11)
C16	0.087 (2)	0.0692 (17)	0.0514 (15)	−0.0145 (16)	0.0036 (14)	−0.0036 (13)
C17	0.0640 (17)	0.0815 (19)	0.0657 (17)	−0.0105 (15)	−0.0056 (14)	0.0130 (15)
C18	0.0521 (14)	0.0751 (18)	0.0782 (19)	−0.0040 (13)	0.0104 (13)	0.0095 (15)
C19	0.0525 (14)	0.0614 (15)	0.0557 (14)	−0.0074 (11)	0.0121 (11)	0.0005 (11)
O1	0.1007 (15)	0.0734 (13)	0.0587 (11)	0.0266 (12)	0.0092 (11)	−0.0091 (10)
O2	0.1251 (19)	0.0873 (15)	0.0560 (11)	0.0263 (14)	0.0186 (12)	−0.0127 (10)

### Geometric parameters (Å, °)

**Table:**

Fe1—C1	2.020 (3)	C7—H7	0.9800
Fe1—C5	2.027 (3)	C8—C9	1.412 (4)
Fe1—C2	2.027 (3)	C8—H8	0.9800
Fe1—C6	2.032 (2)	C9—C10	1.413 (4)
Fe1—C10	2.038 (2)	C9—H9	0.9800
Fe1—C4	2.041 (3)	C10—H10	0.9800
Fe1—C7	2.041 (2)	C11—C12	1.325 (3)
Fe1—C3	2.042 (3)	C11—H11	0.9300
Fe1—C8	2.051 (2)	C12—C13	1.476 (3)
Fe1—C9	2.053 (2)	C12—H12	0.9300
C1—C2	1.388 (6)	C13—O1	1.242 (3)
C1—C5	1.474 (6)	C13—C14	1.471 (3)
C1—H1	0.9800	C14—C19	1.407 (4)
C2—C3	1.352 (5)	C14—C15	1.411 (3)
C2—H2	0.9800	C15—O2	1.346 (4)
C3—C4	1.357 (5)	C15—C16	1.389 (4)
C3—H3	0.9800	C16—C17	1.357 (5)
C4—C5	1.369 (5)	C16—H16	0.9300
C4—H4	0.9800	C17—C18	1.379 (4)
C5—H5	0.9800	C17—H17	0.9300
C6—C7	1.426 (3)	C18—C19	1.374 (4)
C6—C10	1.431 (3)	C18—H18	0.9300
C6—C11	1.453 (3)	C19—H19	0.9300
C7—C8	1.419 (4)	O2—H2A	0.8200
			
C1—Fe1—C5	42.73 (19)	C5—C4—H4	125.0
C1—Fe1—C2	40.12 (18)	Fe1—C4—H4	125.0
C5—Fe1—C2	68.23 (17)	C4—C5—C1	105.9 (4)
C1—Fe1—C6	107.93 (13)	C4—C5—Fe1	70.88 (18)
C5—Fe1—C6	127.61 (14)	C1—C5—Fe1	68.4 (2)
C2—Fe1—C6	121.30 (13)	C4—C5—H5	127.1
C1—Fe1—C10	122.65 (16)	C1—C5—H5	127.1
C5—Fe1—C10	109.42 (13)	Fe1—C5—H5	127.1
C2—Fe1—C10	157.53 (15)	C7—C6—C10	107.5 (2)
C6—Fe1—C10	41.17 (9)	C7—C6—C11	127.2 (2)
C1—Fe1—C4	67.95 (15)	C10—C6—C11	125.2 (2)
C5—Fe1—C4	39.32 (15)	C7—C6—Fe1	69.85 (13)
C2—Fe1—C4	65.85 (15)	C10—C6—Fe1	69.62 (13)
C6—Fe1—C4	164.59 (13)	C11—C6—Fe1	122.83 (16)
C10—Fe1—C4	127.41 (13)	C8—C7—C6	107.6 (2)
C1—Fe1—C7	124.25 (16)	C8—C7—Fe1	70.09 (14)
C5—Fe1—C7	164.50 (16)	C6—C7—Fe1	69.18 (13)
C2—Fe1—C7	107.18 (14)	C8—C7—H7	126.2
C6—Fe1—C7	40.97 (9)	C6—C7—H7	126.2
C10—Fe1—C7	68.79 (10)	Fe1—C7—H7	126.2
C4—Fe1—C7	153.78 (13)	C9—C8—C7	108.7 (2)
C1—Fe1—C3	66.89 (16)	C9—C8—Fe1	69.97 (14)
C5—Fe1—C3	66.53 (15)	C7—C8—Fe1	69.34 (14)
C2—Fe1—C3	38.79 (16)	C9—C8—H8	125.6
C6—Fe1—C3	154.81 (13)	C7—C8—H8	125.6
C10—Fe1—C3	162.56 (13)	Fe1—C8—H8	125.6
C4—Fe1—C3	38.82 (14)	C8—C9—C10	108.0 (2)
C7—Fe1—C3	119.85 (13)	C8—C9—Fe1	69.78 (14)
C1—Fe1—C8	160.64 (19)	C10—C9—Fe1	69.20 (14)
C5—Fe1—C8	154.32 (17)	C8—C9—H9	126.0
C2—Fe1—C8	124.21 (17)	C10—C9—H9	126.0
C6—Fe1—C8	68.40 (10)	Fe1—C9—H9	126.0
C10—Fe1—C8	67.98 (11)	C9—C10—C6	108.1 (2)
C4—Fe1—C8	120.54 (13)	C9—C10—Fe1	70.40 (15)
C7—Fe1—C8	40.57 (10)	C6—C10—Fe1	69.21 (13)
C3—Fe1—C8	107.83 (13)	C9—C10—H10	125.9
C1—Fe1—C9	157.92 (19)	C6—C10—H10	125.9
C5—Fe1—C9	120.96 (16)	Fe1—C10—H10	125.9
C2—Fe1—C9	160.54 (17)	C12—C11—C6	125.4 (2)
C6—Fe1—C9	68.60 (10)	C12—C11—H11	117.3
C10—Fe1—C9	40.39 (10)	C6—C11—H11	117.3
C4—Fe1—C9	109.26 (12)	C11—C12—C13	121.6 (2)
C7—Fe1—C9	68.36 (10)	C11—C12—H12	119.2
C3—Fe1—C9	125.61 (14)	C13—C12—H12	119.2
C8—Fe1—C9	40.25 (10)	O1—C13—C14	120.1 (2)
C2—C1—C5	105.2 (3)	O1—C13—C12	119.6 (2)
C2—C1—Fe1	70.23 (19)	C14—C13—C12	120.3 (2)
C5—C1—Fe1	68.89 (18)	C19—C14—C15	117.3 (2)
C2—C1—H1	127.4	C19—C14—C13	122.9 (2)
C5—C1—H1	127.4	C15—C14—C13	119.8 (2)
Fe1—C1—H1	127.4	O2—C15—C16	118.1 (3)
C3—C2—C1	109.6 (4)	O2—C15—C14	121.2 (2)
C3—C2—Fe1	71.20 (19)	C16—C15—C14	120.6 (3)
C1—C2—Fe1	69.6 (2)	C17—C16—C15	120.2 (3)
C3—C2—H2	125.2	C17—C16—H16	119.9
C1—C2—H2	125.2	C15—C16—H16	119.9
Fe1—C2—H2	125.2	C16—C17—C18	120.7 (3)
C2—C3—C4	109.4 (4)	C16—C17—H17	119.6
C2—C3—Fe1	70.0 (2)	C18—C17—H17	119.6
C4—C3—Fe1	70.52 (19)	C19—C18—C17	120.3 (3)
C2—C3—H3	125.3	C19—C18—H18	119.9
C4—C3—H3	125.3	C17—C18—H18	119.9
Fe1—C3—H3	125.3	C18—C19—C14	120.9 (3)
C3—C4—C5	110.0 (4)	C18—C19—H19	119.6
C3—C4—Fe1	70.66 (18)	C14—C19—H19	119.6
C5—C4—Fe1	69.8 (2)	C15—O2—H2A	109.5
C3—C4—H4	125.0		
			
C5—Fe1—C1—C2	115.9 (3)	C5—Fe1—C6—C11	43.5 (3)
C6—Fe1—C1—C2	−117.6 (2)	C2—Fe1—C6—C11	−41.9 (3)
C10—Fe1—C1—C2	−160.5 (2)	C10—Fe1—C6—C11	119.4 (3)
C4—Fe1—C1—C2	78.2 (2)	C4—Fe1—C6—C11	71.9 (5)
C7—Fe1—C1—C2	−75.3 (3)	C7—Fe1—C6—C11	−122.0 (3)
C3—Fe1—C1—C2	36.0 (2)	C3—Fe1—C6—C11	−73.7 (4)
C8—Fe1—C1—C2	−41.8 (5)	C8—Fe1—C6—C11	−159.8 (2)
C9—Fe1—C1—C2	165.2 (3)	C9—Fe1—C6—C11	156.8 (2)
C2—Fe1—C1—C5	−115.9 (3)	C10—C6—C7—C8	0.1 (3)
C6—Fe1—C1—C5	126.5 (2)	C11—C6—C7—C8	176.3 (2)
C10—Fe1—C1—C5	83.6 (2)	Fe1—C6—C7—C8	59.81 (17)
C4—Fe1—C1—C5	−37.7 (2)	C10—C6—C7—Fe1	−59.67 (16)
C7—Fe1—C1—C5	168.8 (2)	C11—C6—C7—Fe1	116.5 (2)
C3—Fe1—C1—C5	−79.8 (2)	C1—Fe1—C7—C8	163.7 (2)
C8—Fe1—C1—C5	−157.7 (3)	C5—Fe1—C7—C8	−166.8 (5)
C9—Fe1—C1—C5	49.4 (4)	C2—Fe1—C7—C8	122.9 (2)
C5—C1—C2—C3	0.2 (4)	C6—Fe1—C7—C8	−118.8 (2)
Fe1—C1—C2—C3	−60.2 (2)	C10—Fe1—C7—C8	−80.49 (16)
C5—C1—C2—Fe1	60.4 (2)	C4—Fe1—C7—C8	52.9 (3)
C1—Fe1—C2—C3	120.3 (4)	C3—Fe1—C7—C8	82.7 (2)
C5—Fe1—C2—C3	79.2 (3)	C9—Fe1—C7—C8	−36.95 (16)
C6—Fe1—C2—C3	−159.1 (2)	C1—Fe1—C7—C6	−77.5 (2)
C10—Fe1—C2—C3	167.5 (3)	C5—Fe1—C7—C6	−48.0 (5)
C4—Fe1—C2—C3	36.4 (2)	C2—Fe1—C7—C6	−118.3 (2)
C7—Fe1—C2—C3	−116.5 (2)	C10—Fe1—C7—C6	38.31 (14)
C8—Fe1—C2—C3	−75.2 (3)	C4—Fe1—C7—C6	171.7 (3)
C9—Fe1—C2—C3	−43.0 (5)	C3—Fe1—C7—C6	−158.51 (17)
C5—Fe1—C2—C1	−41.1 (3)	C8—Fe1—C7—C6	118.8 (2)
C6—Fe1—C2—C1	80.7 (3)	C9—Fe1—C7—C6	81.85 (15)
C10—Fe1—C2—C1	47.3 (5)	C6—C7—C8—C9	−0.2 (3)
C4—Fe1—C2—C1	−83.9 (3)	Fe1—C7—C8—C9	59.06 (18)
C7—Fe1—C2—C1	123.2 (2)	C6—C7—C8—Fe1	−59.24 (17)
C3—Fe1—C2—C1	−120.3 (4)	C1—Fe1—C8—C9	−164.6 (4)
C8—Fe1—C2—C1	164.5 (2)	C5—Fe1—C8—C9	51.8 (4)
C9—Fe1—C2—C1	−163.3 (3)	C2—Fe1—C8—C9	164.04 (19)
C1—C2—C3—C4	−0.4 (4)	C6—Fe1—C8—C9	−81.97 (16)
Fe1—C2—C3—C4	−59.7 (2)	C10—Fe1—C8—C9	−37.47 (15)
C1—C2—C3—Fe1	59.3 (2)	C4—Fe1—C8—C9	84.0 (2)
C1—Fe1—C3—C2	−37.2 (3)	C7—Fe1—C8—C9	−120.1 (2)
C5—Fe1—C3—C2	−84.0 (3)	C3—Fe1—C8—C9	124.51 (19)
C6—Fe1—C3—C2	45.8 (4)	C1—Fe1—C8—C7	−44.5 (5)
C10—Fe1—C3—C2	−164.0 (4)	C5—Fe1—C8—C7	171.9 (3)
C4—Fe1—C3—C2	−120.3 (3)	C2—Fe1—C8—C7	−75.8 (2)
C7—Fe1—C3—C2	80.2 (3)	C6—Fe1—C8—C7	38.16 (14)
C8—Fe1—C3—C2	122.9 (3)	C10—Fe1—C8—C7	82.67 (16)
C9—Fe1—C3—C2	163.8 (2)	C4—Fe1—C8—C7	−155.85 (18)
C1—Fe1—C3—C4	83.1 (3)	C3—Fe1—C8—C7	−115.35 (18)
C5—Fe1—C3—C4	36.3 (2)	C9—Fe1—C8—C7	120.1 (2)
C2—Fe1—C3—C4	120.3 (3)	C7—C8—C9—C10	0.1 (3)
C6—Fe1—C3—C4	166.1 (2)	Fe1—C8—C9—C10	58.82 (18)
C10—Fe1—C3—C4	−43.7 (5)	C7—C8—C9—Fe1	−58.68 (18)
C7—Fe1—C3—C4	−159.51 (19)	C1—Fe1—C9—C8	166.5 (3)
C8—Fe1—C3—C4	−116.9 (2)	C5—Fe1—C9—C8	−156.6 (2)
C9—Fe1—C3—C4	−75.9 (2)	C2—Fe1—C9—C8	−43.0 (4)
C2—C3—C4—C5	0.5 (4)	C6—Fe1—C9—C8	81.43 (16)
Fe1—C3—C4—C5	−58.9 (2)	C10—Fe1—C9—C8	119.5 (2)
C2—C3—C4—Fe1	59.4 (2)	C4—Fe1—C9—C8	−114.86 (18)
C1—Fe1—C4—C3	−80.1 (3)	C7—Fe1—C9—C8	37.23 (15)
C5—Fe1—C4—C3	−121.0 (3)	C3—Fe1—C9—C8	−74.8 (2)
C2—Fe1—C4—C3	−36.4 (2)	C1—Fe1—C9—C10	47.0 (4)
C6—Fe1—C4—C3	−157.4 (4)	C5—Fe1—C9—C10	83.9 (2)
C10—Fe1—C4—C3	164.9 (2)	C2—Fe1—C9—C10	−162.6 (4)
C7—Fe1—C4—C3	43.4 (4)	C6—Fe1—C9—C10	−38.09 (15)
C8—Fe1—C4—C3	80.4 (2)	C4—Fe1—C9—C10	125.63 (18)
C9—Fe1—C4—C3	123.3 (2)	C7—Fe1—C9—C10	−82.28 (16)
C1—Fe1—C4—C5	40.9 (3)	C3—Fe1—C9—C10	165.73 (17)
C2—Fe1—C4—C5	84.6 (3)	C8—Fe1—C9—C10	−119.5 (2)
C6—Fe1—C4—C5	−36.5 (6)	C8—C9—C10—C6	0.0 (3)
C10—Fe1—C4—C5	−74.1 (3)	Fe1—C9—C10—C6	59.13 (17)
C7—Fe1—C4—C5	164.4 (3)	C8—C9—C10—Fe1	−59.18 (18)
C3—Fe1—C4—C5	121.0 (3)	C7—C6—C10—C9	−0.1 (3)
C8—Fe1—C4—C5	−158.6 (2)	C11—C6—C10—C9	−176.3 (2)
C9—Fe1—C4—C5	−115.7 (3)	Fe1—C6—C10—C9	−59.87 (18)
C3—C4—C5—C1	−0.3 (4)	C7—C6—C10—Fe1	59.81 (16)
Fe1—C4—C5—C1	−59.7 (2)	C11—C6—C10—Fe1	−116.5 (2)
C3—C4—C5—Fe1	59.4 (2)	C1—Fe1—C10—C9	−160.9 (2)
C2—C1—C5—C4	0.1 (4)	C5—Fe1—C10—C9	−115.3 (2)
Fe1—C1—C5—C4	61.4 (2)	C2—Fe1—C10—C9	164.8 (4)
C2—C1—C5—Fe1	−61.3 (2)	C6—Fe1—C10—C9	119.3 (2)
C1—Fe1—C5—C4	−116.6 (3)	C4—Fe1—C10—C9	−75.0 (2)
C2—Fe1—C5—C4	−78.0 (3)	C7—Fe1—C10—C9	81.12 (16)
C6—Fe1—C5—C4	168.50 (19)	C3—Fe1—C10—C9	−42.0 (5)
C10—Fe1—C5—C4	125.9 (2)	C8—Fe1—C10—C9	37.34 (15)
C7—Fe1—C5—C4	−153.6 (4)	C1—Fe1—C10—C6	79.8 (2)
C3—Fe1—C5—C4	−35.9 (2)	C5—Fe1—C10—C6	125.4 (2)
C8—Fe1—C5—C4	46.5 (4)	C2—Fe1—C10—C6	45.6 (4)
C9—Fe1—C5—C4	82.8 (3)	C4—Fe1—C10—C6	165.71 (16)
C2—Fe1—C5—C1	38.6 (2)	C7—Fe1—C10—C6	−38.13 (14)
C6—Fe1—C5—C1	−74.9 (3)	C3—Fe1—C10—C6	−161.2 (4)
C10—Fe1—C5—C1	−117.5 (2)	C8—Fe1—C10—C6	−81.91 (16)
C4—Fe1—C5—C1	116.6 (3)	C9—Fe1—C10—C6	−119.3 (2)
C7—Fe1—C5—C1	−36.9 (6)	C7—C6—C11—C12	8.4 (4)
C3—Fe1—C5—C1	80.8 (3)	C10—C6—C11—C12	−176.1 (2)
C8—Fe1—C5—C1	163.1 (3)	Fe1—C6—C11—C12	96.9 (3)
C9—Fe1—C5—C1	−160.6 (2)	C6—C11—C12—C13	−178.5 (2)
C1—Fe1—C6—C7	122.0 (2)	C11—C12—C13—O1	0.1 (4)
C5—Fe1—C6—C7	165.5 (2)	C11—C12—C13—C14	177.5 (2)
C2—Fe1—C6—C7	80.0 (2)	O1—C13—C14—C19	−177.5 (2)
C10—Fe1—C6—C7	−118.6 (2)	C12—C13—C14—C19	5.2 (4)
C4—Fe1—C6—C7	−166.1 (4)	O1—C13—C14—C15	3.2 (4)
C3—Fe1—C6—C7	48.3 (3)	C12—C13—C14—C15	−174.2 (2)
C8—Fe1—C6—C7	−37.80 (15)	C19—C14—C15—O2	179.5 (2)
C9—Fe1—C6—C7	−81.21 (15)	C13—C14—C15—O2	−1.1 (4)
C1—Fe1—C6—C10	−119.4 (2)	C19—C14—C15—C16	−2.2 (4)
C5—Fe1—C6—C10	−75.9 (2)	C13—C14—C15—C16	177.2 (2)
C2—Fe1—C6—C10	−161.4 (2)	O2—C15—C16—C17	−179.1 (3)
C4—Fe1—C6—C10	−47.5 (5)	C14—C15—C16—C17	2.6 (4)
C7—Fe1—C6—C10	118.6 (2)	C15—C16—C17—C18	−1.3 (5)
C3—Fe1—C6—C10	166.9 (3)	C16—C17—C18—C19	−0.4 (5)
C8—Fe1—C6—C10	80.80 (16)	C17—C18—C19—C14	0.7 (4)
C9—Fe1—C6—C10	37.39 (15)	C15—C14—C19—C18	0.6 (4)
C1—Fe1—C6—C11	0.0 (3)	C13—C14—C19—C18	−178.8 (2)

### Hydrogen-bond geometry (Å, °)

**Table:**

*D*—H···*A*	*D*—H	H···*A*	*D*···*A*	*D*—H···*A*
O2—H2A···O1	0.82	1.79	2.523 (3)	148
